# Study of Spin–Orbit Interactions and Interlayer Ferromagnetic Coupling in Co/Pt/Co Trilayers in a Wide Range of Heavy-Metal Thickness

**DOI:** 10.1021/acsami.1c11675

**Published:** 2021-09-24

**Authors:** Piotr Ogrodnik, Krzysztof Grochot, Łukasz Karwacki, Jarosław Kanak, Michał Prokop, Jakub Chȩciński, Witold Skowroński, Sławomir Ziȩtek, Tomasz Stobiecki

**Affiliations:** † Institute of Electronics, AGH University of Science and Technology, 30-059 Kraków, Poland; ‡ Institute for Theoretical Physics, Utrecht University, Princetonplein 5, 3584 CC Utrecht, The Netherlands; § Catalan Institute of Nanoscience and Nanotechnology (ICN2), CSIC and BIST, Campus UAB, Bellaterra, 08193 Barcelona, Spain; ∥ Faculty of Physics, Warsaw University of Technology, 00-662 Warsaw, Poland; ⊥ Faculty of Physics and Applied Computer Science, AGH University of Science and Technology, 30-059 Kraków, Poland; # Institute of Molecular Physics, Polish Academy of Sciences, ul. M. Smoluchowskiego 17, 60-179 Poznań, Poland

**Keywords:** ferromagnetic resonance, spin Hall effect, magnetoresistance, spin−orbit torques, Rashba−Edelstein effect

## Abstract

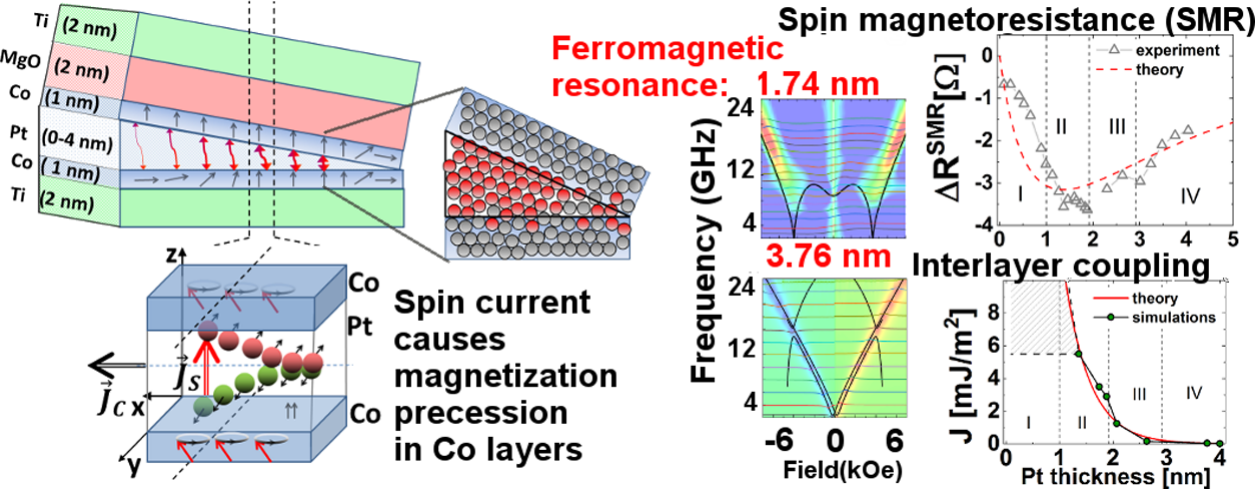

The spin–orbit torque, a torque induced by a charge current flowing through the heavy-metal-conducting layer with strong spin–orbit interactions, provides an efficient way to control the magnetization direction in heavy-metal/ferromagnet nanostructures, required for applications in the emergent magnetic technologies like random access memories, high-frequency nano-oscillators, or bioinspired neuromorphic computations. We study the interface properties, magnetization dynamics, magnetostatic features, and spin–orbit interactions within the multilayer system Ti(2)/Co(1)/Pt(0–4)/Co(1)/MgO(2)/Ti(2) (thicknesses in nanometers) patterned by optical lithography on micrometer-sized bars. In the investigated devices, Pt is used as a source of the spin current and as a nonmagnetic spacer with variable thickness, which enables the magnitude of the interlayer ferromagnetic exchange coupling to be effectively tuned. We also find the Pt thickness-dependent changes in magnetic anisotropies, magnetoresistances, effective Hall angles, and, eventually, spin–orbit torque fields at interfaces. The experimental findings are supported by the relevant interface structure-related simulations, micromagnetic, macrospin, as well as the spin drift-diffusion models. Finally, the contribution of the spin–orbital Edelstein–Rashba interfacial fields is also briefly discussed in the analysis.

## 1 Introduction

The magnetic multilayer structures consisting of thin ferromagnetic (F) layers and nonmagnetic spacers are known to exhibit plenty of phenomena, among which one can find those extensively studied for the last decades like anisotropic, giant and tunneling magnetoresistance or spin-transfer torque effect (STT),^1,2^ and recently, current-driven spin–orbit torque (SOT) magnetization switching.^3^ These effects are widely exploited in the spintronic devices, magnetic random access memories (MRAM) like STT-MRAM and SOT-MRAM,^4−7^ as well as may be exploited in magnetic sensors (including magnetic nanoparticles) and nano-oscillators.^8,9^ Such devices include nonmagnetic layers that are crucial for their features and performance. These layers may be both insulating (e.g., MgO in magnetic tunnel junctions) and metallic (e.g., Cu, Au in GMR devices).^9^ Recently, the nonmagnetic layers made of heavy metallic (HM) elements (W, Ta, Pt, and their alloys^10−12^) are extensively studied because of their large spin–orbit coupling (SOC).^13^ Such layers combined with ferromagnetic ones (typically Co, CoFeB) are expected to have new spin transport properties related to the SOC, e.g., spin Hall effect (SHE) and Rashba–Edelstein effect (REE).^14,15^ Although the SHE occurs in a single HM layer,^16^ it is detectable in heterostructures with ferromagnets only, such as F/HM bi-^17^ and F/HM/F trilayers.^18,19^ In these structures, the spin-polarized electrons can accumulate at the HM/F interfaces and then may be efficiently injected into the F layer exerting the spin–orbit torque (SOT) on its magnetic moment. This effect has been predicted theoretically^20−22^ and reported in many experimental works on SOT-induced magnetic dynamics^23^ and magnetic switching.^19,24−26^ Especially for the F layers with a magnetic perpendicular anisotropy, the SOT enables a promising way to design efficient, ultralow power consumption spintronic devices. Apart from the SHE and related spin accumulation, the other pure interfacial effects, like charge-spin conversion REE at interfaces, contribute to the SOT.^27−29^ Particularly, in multilayer systems with the symmetry-breaking axis along the direction of the current flow, the REE enables field-free magnetization switching.^30,31^ Similar effect was also found in the magnetic multilayers in the presence of spin current gradients.^32,33^ Therefore, interface engineering and quantifying the REE become significant for the optimization of SOT-based devices.^34−36^ The spin currents injected into the F layer and SOTs may be examined by electric measurements through its magnetoresistance^37−39^ and the anomalous Hall effect (AHE).^40^ The change of the resistance of the hybrid structure caused by the above effects is referred to as spin Hall magnetoresistance (SMR).^41^ Up to date, the specific multilayer structures (bi- and trilayers) were studied in detail. Among them, we find CoFeB-based structures like W/i-CoFeB/Pt^42^ and p-CoFeB/Ta, as well as the Co-based multilayers like^43^ p-Co/Pt/i-Co,^19^ p-Co/Pt,^44−46^ i-Co/Ta,^46^ Ta/i-Co/Pt,^47^ Ru/p-Co/Ru, and Ru/p-Co/Ru/W,^48^ where p(i) stands for perpendicular(in-plane) anisotropy. Also, the recent studies on Pt/Co/Ru/Co/Pt showed that the RKKY interlayer exchange coupling (IEC) could tailor the properties of the multilayers.^49^

In this paper, we present the detailed studies of the Co/Pt/Co system with the use of the electrically detected FMR (ferromagnetic resonance), as well as low-frequency harmonic Hall voltage and static magnetotransport measurements. Here, the Pt layer plays a double role in the considered structure, first as a source of substantial spin currents and second as an essential element of the exchange ferromagnetic coupling mechanism. Therefore, the Pt thickness can be varied to control the spin currents and interlayer coupling, both essential for designing SOT-MRAM and high-frequency spintronic devices. We provide the results on the resonance frequencies and the SOT effective fields depending on the Pt thickness. Also, we analyze the magnetic parameters of the system like anisotropies, saturation magnetizations, and the IEC. We show that anisotropies and the IEC strongly depend on the Pt thickness, particularly for Pt layer thicknesses less than 2 nm. For such a thin Pt, the transition from the effective in-plane Co anisotropy to the perpendicular one may occur. We account for the features by providing reliable theoretical macrospin models of magnetization dynamics, magnetoresistance, and the effective spin Hall effect angle.

## 2 Experimental Section

### 2.1 Multilayer Stack

**Figure 1:**
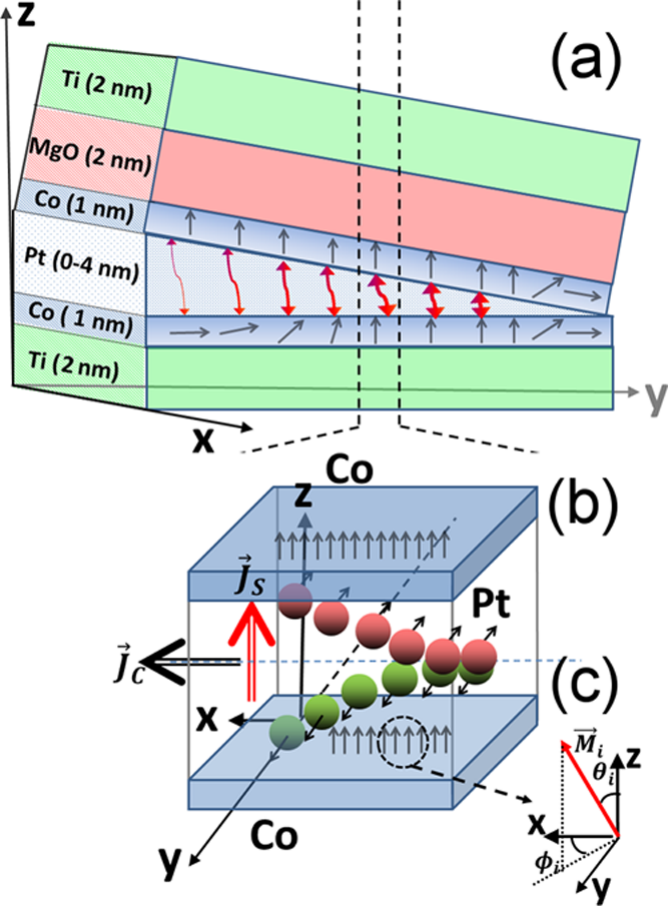
(a) Experimental multilayer stack with a wedge of Pt. The red thick (thin) wavy arrows indicate strong (weak) IEC, whereas the gray arrows show the change in the magnetization alignment with the Pt thickness; (b) the patterned device for a certain thickness of Pt—the arrows indicate the direction of the current flow (*j⃗*_c_) and associated spin current (*j⃗*_s_) due to SHE. The short arrows depicted in the Co layers denote their magnetization vectors for a given Pt thickness at remanence; and (c) the polar and azimuthal angles describing the magnetization direction within the Co layers.

Multilayers are deposited on thermally oxidized Si substrates using magnetron sputtering at room temperature. We study the Co/Pt/Co trilayer within the Ti(2)/Co(1)/Pt(0–4)/Co(1)/MgO(2)/Ti(2) structure shown in Figure 1 (the numbers in parentheses indicate the nominal thickness of the individual layers in nanometers). The Co/Pt/Co trilayer was designed so that it allows us to study the influence of the Pt thickness on the magnetic anisotropy of bottom and top Co layers, the IEC between Co layers through the Pt spacer, magnetization dynamics, and SHE-driven SOT acting on the F layers. For this purpose, both bottom and top thin Co layers should have small anisotropy (differing by interfaces Ti/Co and Co/MgO), with values close to the transition from in-plane to perpendicular. The Ti underlayer improves subsequent layers’ adhesion and smoothens the substrate surface. Moreover, as shown in ref (50), the Ti/Co interface is alloyed due to mixing during magnetron deposition, while the Co/MgO interface is sharp.^51^ Therefore, the top Co layer is characterized by a higher interface perpendicular anisotropy.

### 2.2 Structural Characterization

**Figure 2:**
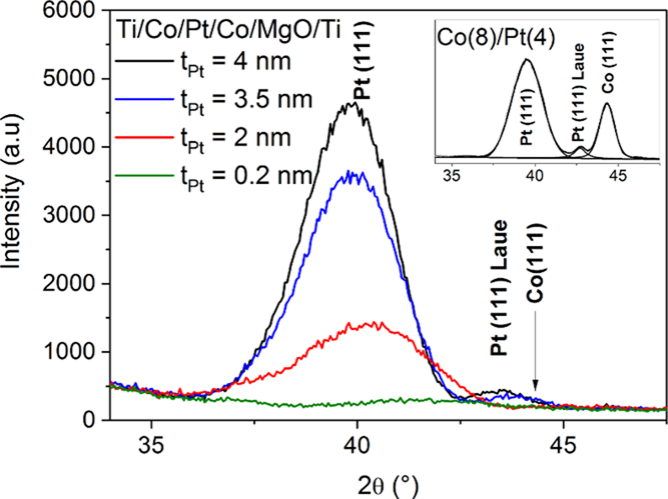
XRD θ–2θ profiles of a Si/SiO_2_/Ti/Co(1)/Pt(0–4)/Co/MgO/Ti measured at different positions of the Pt wedge. The arrow indicates the 2θ position of the structural Co (111) peak visible in the reference sample with 8 nm of Co and 4 nm of the Pt layer thickness (inset).

High-resolution X’Pert–MPD diffractometer with a Cu anode was used for X-ray diffraction (XRD) characterization. Figure 2 shows the XRD θ–2θ profiles of the Si/SiO_2_/Ti/Co(1)/Pt(0–4)/Co(1)/MgO/Ti multilayer measured at different positions of the Pt wedge. The θ–2θ measurements show the preferred growth of the Pt/Co in the [111] direction of the fcc structure. The peak of the Co layers is invisible because of their tiny thicknesses (*t*_Co_ ≈ 1 nm). The arrows indicate the Co (111) peak position present in the thick Co layer case (see the Supplemental Material in refs (19, 52)). The peak on the right side of the Pt(111) is a Laue satellite^19^ that confirms the asymmetry in top Pt/Co and bottom Co/Pt interfaces. The intensity of the profiles depends on the number of Pt atoms. Therefore, in the case of very thin (0.2 nm) Pt layers, the Laue peaks are out of detection of the experimental method. However, one can see that the Pt peak slightly shifts to the right for thin Pt layers. It suggests that most of the Pt layer becomes mixed with Co atoms, making a rather Co–Pt compound than a well-separated layer.

**Figure 3:**
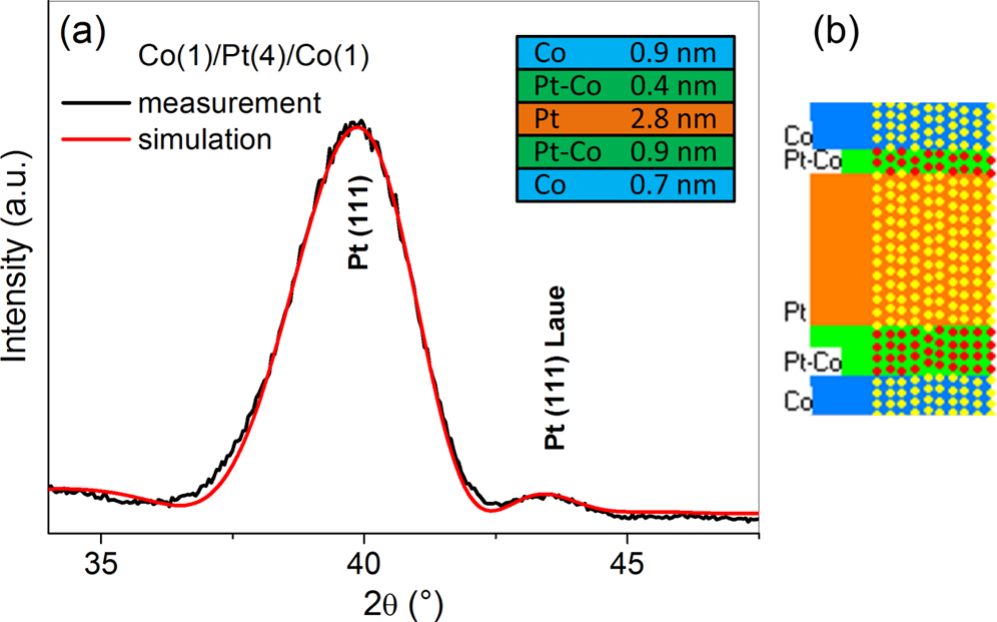
Measured and calculated XRD θ −2θ profiles (a). The assumed thicknesses of the Pt and Co layers and transition area with the Pt–Co mixed interfaces (inset). A snapshot of the Monte Carlo simulation of the interface structure in Co/Pt/Co (b).

Figure 3a shows the profile measured for the Si/SiO_2_/Ti(2)/Co(1)/Pt(4)/Co(1)/MgO(2)/Ti(2) at a Pt thickness of ≈ 4 nm, together with the profile calculated using the simulated^52^ structure (Figure 3b). An excellent agreement between the experimental and theoretical profiles is achieved. The structure was simulated with the assumption of Pt and Co mixing at the interfaces. The simulations represent the columnar grains in the Pt and Co layers and a transition area with the Pt–Co mixed interface. Mixing of the Pt and Co atoms at the interface causes decreasing Pt lattice plane spacing compared to that of pure Pt.

The simulation assumes more significant mixing at the bottom side of the Pt than at the top one. In the former case, the heavy Pt atoms can penetrate the Co layer more easily than in the latter case. Moreover, for Pt within Co, the interfacial enthalpy is −33 kJ/(mole of atoms), whereas, for Co in Pt, the interfacial enthalpy is −26 kJ/(mole of atoms). Higher negative enthalpy results in easier mixing at the bottom Co/Pt interface.

## 3 Theory

### 3.1 Resonance Model

This subsection presents the macrospin model that allows us to calculate resonance frequencies of the considered Co/Pt/Co structure. Since the Co layers may be either coupled or uncoupled, we employ the approach that has been already presented in detail and successfully applied in our previous work.^53^ We describe magnetic moments of each layer by spherical angles (polar θ_*i*_ and azimuthal ϕ_*i*_)
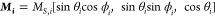
1where *i* = 1(2) is referred to as the top(bottom) cobalt layer. The magnetization dynamics of the system is described by two coupled Landau–Lifshitz–Gilbert (LLG) equations
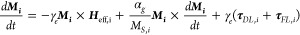
2where  is the gyromagnetic ratio, and α_*g*_ is the Gilbert damping parameter for each layer.

The terms τ_DL_ = *H*_DL_(***m***_*i*_ × ***m***_*i*_ × ***e*^**_*y*_) and τ_FL_ = *H*_FL_(***m***_*i*_ × ***e*^**_*y*_) stand for SOT damping-like (DL) and field-like(FL) components with the unit vector  and the amplitudes *H*_DL_ and *H*_FL_, respectively.

The effective field (*H*_eff_) can be expressed as a functional derivative of the following total magnetic energy of the system
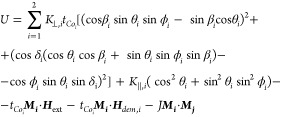
3The complex expression for the anisotropies originates from the rotation of the easy axes around x and y directions with the use of the relevant Euler rotation matrices. The angles β and δ have been introduced to account for a small deviation of the perpendicular anisotropies (*K*_⊥,*i*_) from the perpendicular (*z*) direction (δ, β ≪ π/2). The perpendicular anisotropy terms simplify into a well-known form *K*_⊥,*i*_ sin^2^θ_*i*_ when δ_*i*_, β_*i*_ = 0. Also, we have added a small in-plane contribution *K*_∥_ ≪ *K*_⊥_ along the *y* direction. As long as they are small, they slightly improve the fitting of the macrospin model to the experimental data. In eq 3, *t*_Co,*i*_, ***H***_ext_, ***H***_dem,*i*_, and *J* stand for magnetic layer thickness, external magnetic field, demagnetizing field, and IEC, respectively.

The LLG equation (eq 2) in polar coordinates can be written in the general form
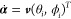
4where α̇ and ***v*** are the vectors containing the spherical angles (θ_1,2_, ϕ_1,2_), time-derivatives, and the right-hand side (RHS) of the LLG equation, respectively. After linearization of ***v*** with respect to small deviations in θ_*i*_ and ϕ_*i*_ from their stationary values, one can write eq 4 in the form
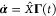
5where *X̂* is a 4 × 4 matrix consisting of the derivatives of the RHS of eq 4 with respect to the angles θ_*i*_, ϕ_*i*_ (i.e., ), while Γ(*t*) = (δα_1_(*t*), ..., δ α_4_(*t*))^*T*^ is a vector containing time-dependent angle differentials, i.e., δα_1_(*t*) ≡ δ θ_1_(*t*), δα_2_(*t*) ≡ δ ϕ_1_(*t*), etc. When small oscillations are assumed and in the absence of the external driving force (i.e., SOT or Oersted field), eq 5 can be rewritten as an eigenvalue problem of the matrix *X̂*
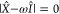
6The solution of the problem provides the complex eigenvalues ω_*i*_ determining two distinct natural resonance angular frequencies of the system, ω_*R*,*i*_ = Re ω_*i*_.

### 3.2 Diffusive Model of the Magnetoresistance

The average longitudinal resistance of our trilayer stack is, in general, dependent on the orientation of magnetizations ***m***_1(2)_ in both ferromagnetic layers and reads
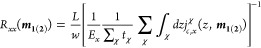
7where *E*_*x*_ is the electric field in the *x* direction, *L* is the length, *w* is the width, and *t*_χ_ is the thickness of layer χ = *HM*, *F*1, *F*2, and ∫_χ_ denotes the integral with limits corresponding to the position of layer χ in the stack. For Pt, i.e., for χ = *HM*, the charge current density reads
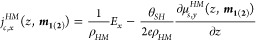
8where ρ_*HM*_ is the resistivity of the Pt layer, θ_*SH*_ is the spin Hall angle defined as the charge-to-spin current conversion efficiency at a very thick HM layer limit, and μ_*s*,*y*_^*HM*^(*z*, ***m***_**1(2)**_) is the spin accumulation, while for the ferromagnetic layers, i.e., χ = *F*1 (χ = *F*2)
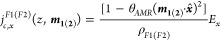
9where ρ_*F*1(*F*2)_ is the resistivity of the corresponding ferromagnetic layer, θ_*AMR*_ is the AMR in the thick ferromagnetic limit (assumed for simplicity the same in both ferromagnetic layers). For more details, see, e.g., ref (37).

To obtain spin accumulation in the Pt layer, we consider the spin current density flowing in Pt
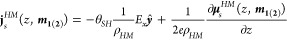
10along with the boundary conditions
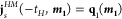
11a
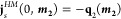
11b
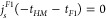
11c
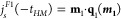
11d
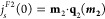
11e
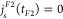
11fwhere the spin current in ferromagnetic metals assumes the following form
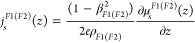
12and the interfacial spin currents
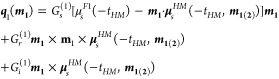
13a
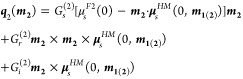
13bwhere *G*_*s*_^(1)^ and *G*_*s*_^(2)^ are spin conductances and *G*_*r*(*i*)_^(1)^ and *G*_*r*(*i*)_^(2)^ are the real (imaginary) parts of spin-mixing conductances for interfaces 1 (F1/HM) and 2 (HM/F2), respectively. Moreover, the effective fields, *H*_DL_ and *H*_FL_ (cf. Section 3.1), due to SHE and spin accumulation at the interfaces can be expressed as follows
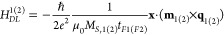
14and
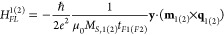
15To fit the appropriate magnetoresistance relations obtained from eq 7, we use the following parameters:^37,54−56^ ρ_HM_ = 59 μΩ cm, ρ_*F*1(*F*2)_ = 72.5 μΩ cm, λ_HM_ = 1.8 nm, λ_*F*1_ = λ_*F*2_ = 7 nm, θ_SH_ = 8%, θ_AMR_ = 0.15%, β_1_ = β_2_ = 0.3, *G*_*s*_^(1)^ = *G*_*s*_^(2)^ = *G*_*r*_^(1)^ = *G*_*i*_^(1)^ = 10^15^ Ω^–1^ m^–2^, and *G*_*r*_^(2)^ = *G*_*i*_^(2)^ = 0.4 *G*_*r*_^(1)^. The parameters were also used to calculate SOT effective fields that turn out to be pivotal in the interpretation of the experimental data presented in Section 4.3.

## 4 Results

### 4.1 FMR and Interlayer Coupling

**Figure 4:**
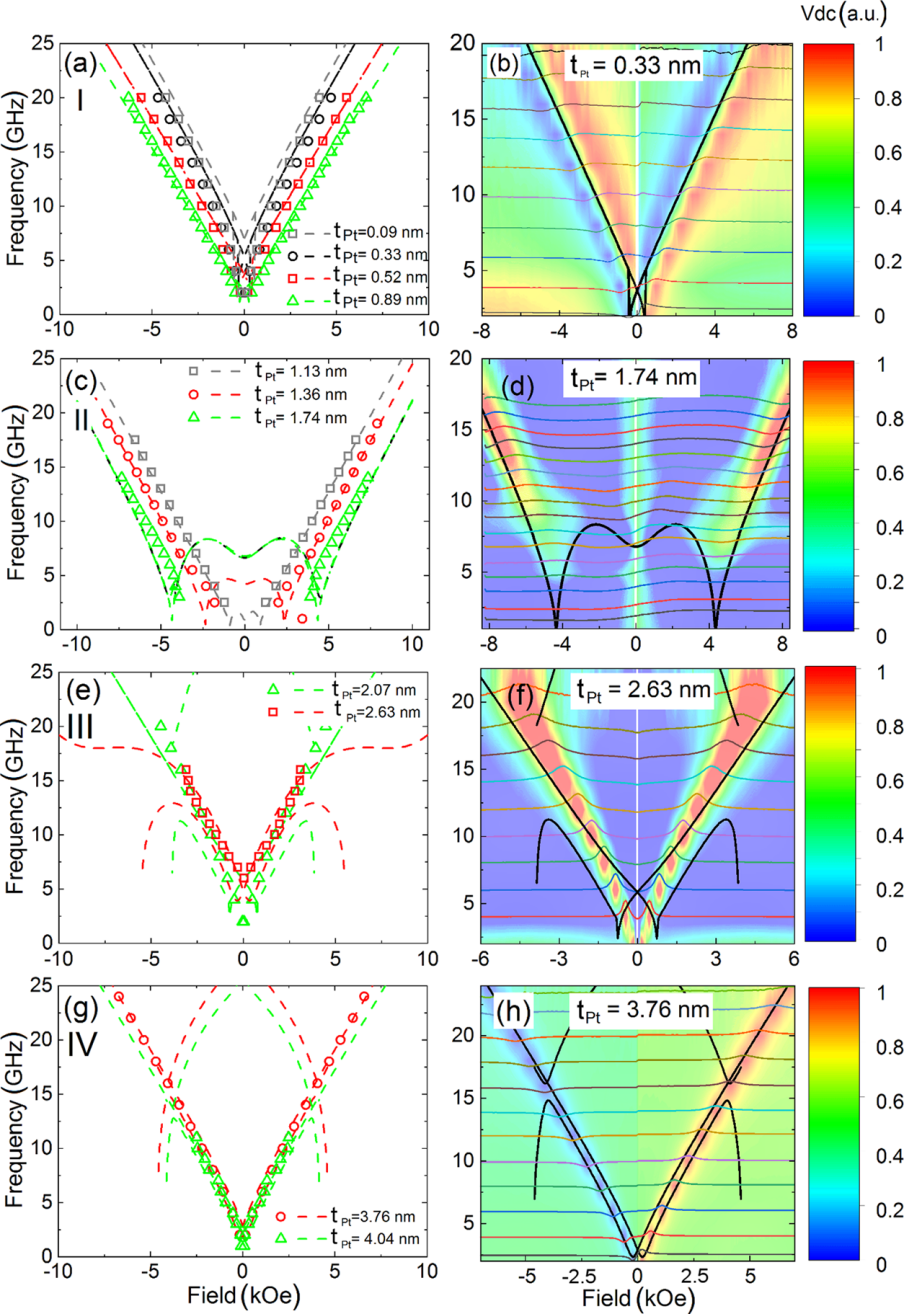
Experimental versus theoretical relations of dispersion for samples from regions I (a, b), II (c, d), III (e, f), and IV (g, h). Left column: the sets of theoretical (lines) and experimental (points) dependencies for each region. Right column: the experimental *V*_DC_ spectra shown as color map (color is the magnitude of the SD signal) and the source raw spectra measured at the frequency ranging from 2 to 20 GHz (light color lines), with the corresponding theoretical *f*(*H*) dependencies (solid black lines). The macrospin simulation magnetic parameters are the same as presented in Figure 5a–d.

First, we measured the magnetization dynamics of the Co/Pt/Co sample in a wide range of Pt thickness from strong through moderate coupling to completely decoupled Co layers. The dynamics was investigated using the electrically detected FMR through the spin diode effect,^57^ as described in Section 6.3. We observed the dispersion relations changing once the Pt thickness reaches boundary values. Thus, we could point out the distinct regions where the system behaves differently. This feature is illustrated in Figure 4. On the right panel of Figure 4, one can see the color FMR spectral line shapes. On the left panel, points correspond to the experimental resonance frequencies. On both sides of this figure, we show that for thin Pt spacer (below 1 nm), the dispersion relations are typical Kittel-like dependencies and move toward lower frequencies when the *t*_*Pt*_ is growing. Next, for *t*_Pt_ > 1 *nm*, the *f*(*H*_*r*_) changes their slopes. Also, their branches part from each other, especially at low frequencies when a sort of resonance mode gaps in experimental data occurs. The increase of the Pt thickness (*t*_*Pt*_ > 2 nm) provides the Kittel-like dependencies again. However, for thick Pt (*t*_Pt_ > 3 nm), the experimental *f*(*H*_*r*_) practically does not change anymore with the Pt thickness.

To understand the *f*(*H*_*r*_) dependence on the Pt thickness, we performed the macrospin simulations and a vast number of fittings for the whole range of the Pt thickness, i.e., from 0.09 to 4.04 nm (Figure 4). Varying the anisotropies (*K*_⊥(∥),1(2)_), perpendicular easy-axis deviation angles (β_1(2)_,δ_1(2)_), magnetization saturations (*M*_*S*1(2)_), and the interlayer coupling strength (*J*) from eq 3, we reproduced the difference in the dynamical behavior of the structure and were able to identify the boundaries between different regions of Pt thickness where these behaviors occur, namely, regions I (thin Pt), II (medium Pt), III (intermediate Pt), and IV (thick Pt). Despite the similarity of *f*(*H*_*r*_) in regions III and IV, we refer to the former as the intermediate since the differences occur in magnetoresistance results discussed in the further part of the paper. The presence of the additional modes (especially in regions III and IV) that were not registered in the experiment can be explained in a couple of ways. First, the theoretical results based on the model presented in Section 3.1 come from the solution of the eigenvalue problem, i.e., the model predicts all possible steady-state modes, regardless of the source of their excitations. On the other hand, the different way of forcing the excitation (by SOTs or Oersted field) is inherent. The resonance modes are not always excited, depending on the force amplitude and its origin. Second, these additional modes are related to the independent dynamics of two magnetizations of F1 and F2 layers due to weak interlayer coupling in regions III and IV. In contrast to regions I and II, there are no collective oscillations and therefore the mode with the large amplitude originates from the in-plane, whereas all other modes come from the perpendicular magnetization dynamics. However, the magnetization oscillations in the latter case do not contribute to the SD signal because of the significant effective damping caused by spin-pumping.^58^

**Figure 5:**
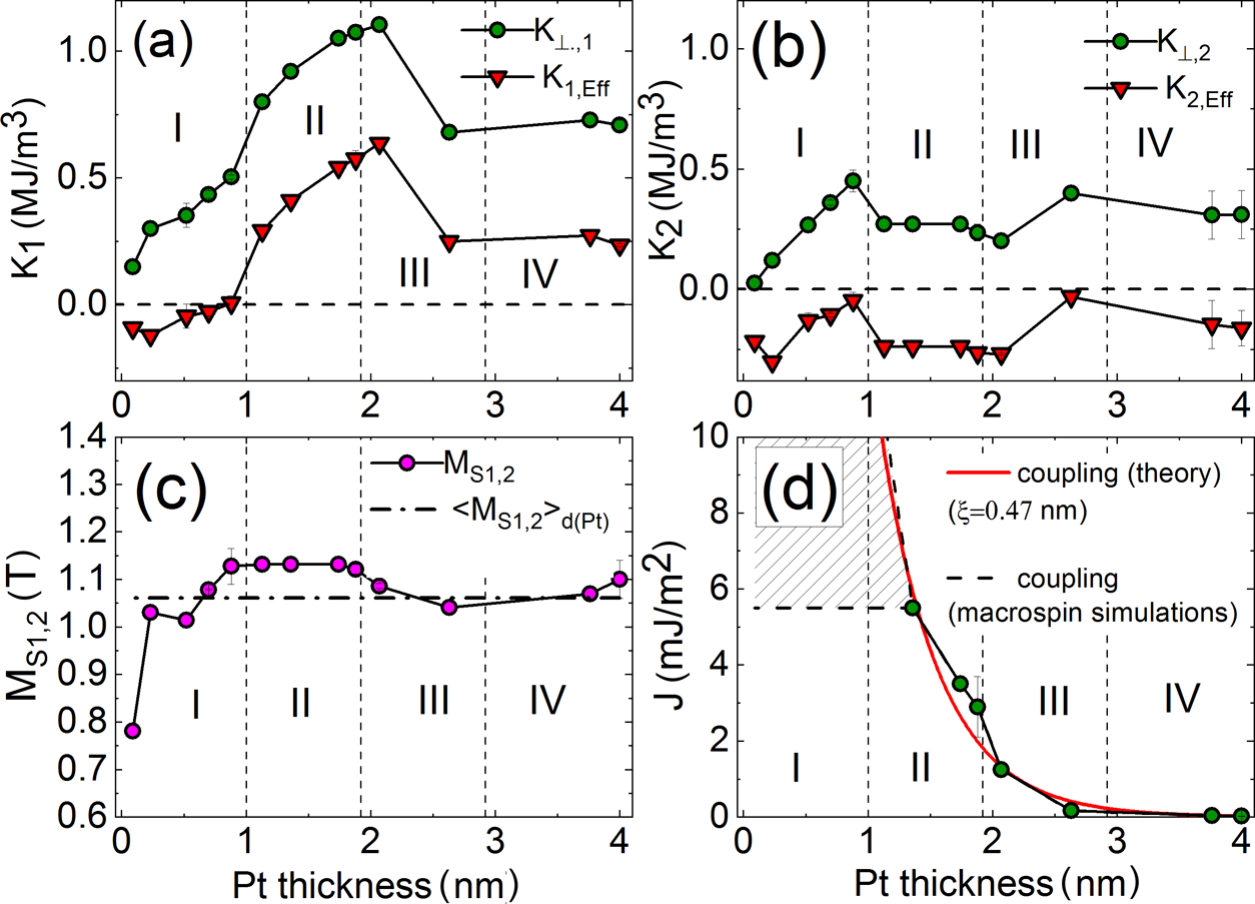
Magnetic parameters of the Co layers as a function of the Pt layer thickness derived from the macrospin simulations of the spin diode FMR spectra: perpendicular and effective anisotropies (a, b), magnetization saturation (c), and interlayer coupling (d).

**Figure 6:**
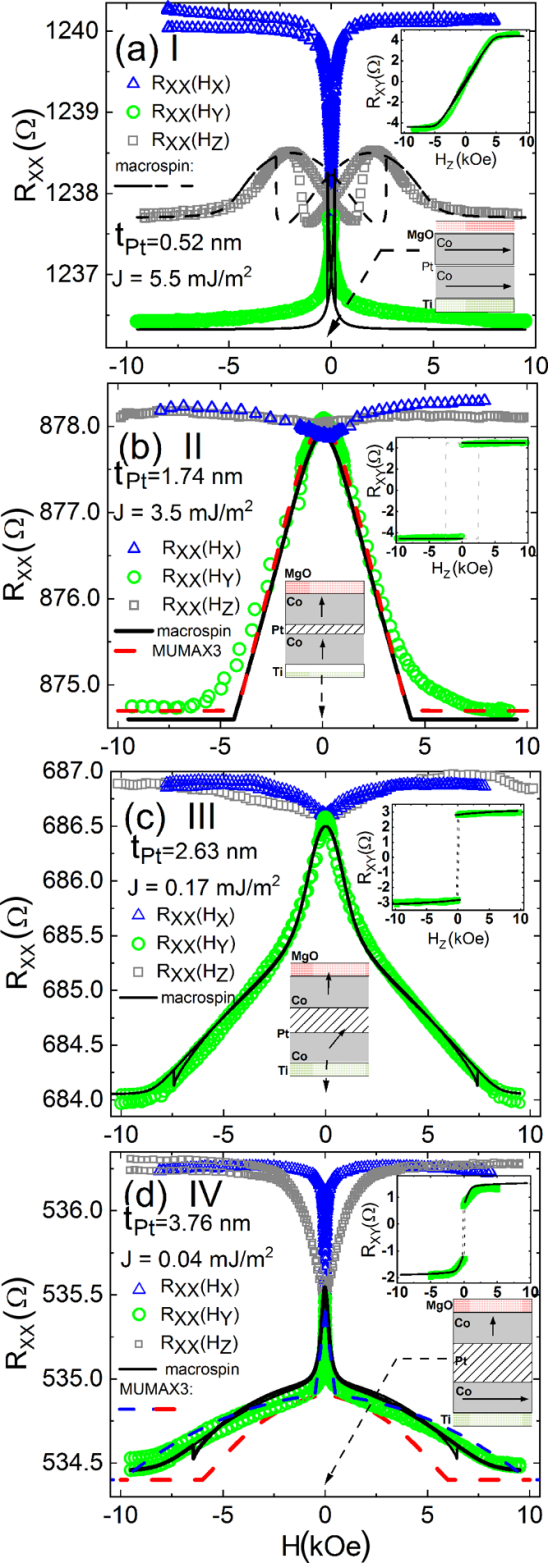
Magnetoresistances *R*_*xx*_ and *R*_*xy*_ (inset) as a function of the magnetic field in the samples with Pt thickness: (a) 0.52 nm as an example from region I, (b) 1.74 nm from region II, (c) 2.63 nm from region III, and (d) 3.76 nm from region IV: *R*_*xx*_ experimental data measured at the magnetic field applied in *x* (blue triangles), *y* (green circles), and *z* (gray squares) directions. The depicted diagrams for all regions indicate the direction of magnetizations of magnetic layers at remanence. The macrospin (black solid and dashed lines) and micromagnetic (dashed red and blue lines) simulations of *R*_*xx*_(*H*_*y*_). Micromagnetic simulations for *t*_Pt_ = 3.76 nm were performed using the same parameters (cf. Figure 5) as derived from the macrospin model (red-dashed line), as well as for a *K*_1_ anisotropy increased by 0.17 MJ/m^3^ (blue dashed line).

The macrospin parameters are summarized in Figure 5. The vertical dashed lines indicate the boundaries between regions I, II, III, and IV. Figure 5a–d presents perpendicular anisotropies *K*_⊥,1,2_, saturation magnetization *M*_*S*,1,2_, as well as the strength of the interlayer coupling. We also depicted the effective anisotropies . The in-plane anisotropies (see the Supporting Information) have small values that have the importance in reproducing subtle *R*_*xx*_ (*H*_*z*_) dependencies for the thinnest Pt layers only (e.g., see in Figure 6a). On the other hand, the anisotropy deviation angles (less than 30°) had to be introduced so that we could find a set of magnetic parameters reproducing both the static (magnetoresistance, AHE) and dynamic (FMR) characteristics simultaneously. One can see that the Co layer (indexed as 1) covered by the MgO layer exhibits a larger perpendicular anisotropy than that adjacent to the Ti layer (indexed as 2), similarly as in the system Si/SiO2/Ti(2)/Co(3)/Pt(t_*Pt*_)/Co(1)/MgO(2)/Ti(2) examined in our previous work.^19^ Moreover, on the basis of magnetization measurements in an external perpendicular field, using vibrating sample magnetometer (VSM), we showed that the sample Pt(4)/Co(1)/MgO(2)/Ti(2) has a smaller effective anisotropy field (*H*_K,eff_ = 1.3 kOe) than Ti(2)/Co(1)/Pt(4) (*H*_K,eff_ = 1.65 kOe). In Figure 5a,b, we show that the effective anisotropy *K*_eff,1_ changes its sign, while the *K*_eff,2_ is negative for all Pt thicknesses. The change in the sign of the effective anisotropy is related to the boundary between regions I and II. Furthermore, one can see that *K*_⊥,1_ increases with the Pt thickness up to *t*_Pt_ = 2 nm, whereas the value of *K*_⊥,2_ is growing just up to 1 nm. Above this thickness, *K*_⊥,2_ is rather stable, and its values are more or less 0.2 MJ/m^3^. The *K*_⊥,1_ reaches its highest value at *t*_Pt_ ≈ 2 nm when it drops to the level of about 0.75 MJ/m^3^. We relate the different values of *K*_⊥,1_ than those of *K*_⊥,2_ to the much more efficient perpendicular anisotropy at Co/MgO than at the Co/Ti interface. On the other hand, the mean value magnetization saturation averaged over the whole range of the Pt thickness is about 1.07 T for both Co layers. The actual value for a given *t*_Pt_ differs by ±10 %. The abrupt decrease in *M*_S_ for the very thin Pt layer (0.09 nm) is caused by the quality of the interfaces and related intermixing of Pt and Co atoms.

The last but not least, the parameter derived from the macrospin is the interlayer coupling energy *J*. The polarization of the Pt is the mechanism of the interaction between two magnetic moments in Co layers. Such an interaction is ferromagnetic by its very nature,^59^ whereas the dipolar coupling (neglected here) is antiferromagnetic. The indirect way to probe the coupling (and the polarization of the Pt) by electrical detection is to measure FMR by rectification of radiofrequency current.^60−62^ We derived the coupling (*J*) from the macrospin simulations, similarly to magnetizations and anisotropies. The coupling dependence on Pt thickness is shown in Figure 5d. However, we could estimate *J* down to a Pt thickness of 1.36 nm, at which *J* = 5.5 mJ/m^2^. Below this thickness, the coupling has no effect on the resonance fields at frequencies experimentally accessible (<25 GHz). The strong coupling causes both Co layers to rotate in the same manner, and they can be treated as one layer rather than two separate layers. In addition, two magnetizations of Co layers oscillate in phase (acoustic mode). It is seen in the experiment as the observed low-frequency mode.^53^ On the contrary, the magnetization oscillations with opposite phases correspond to the high-frequency optical mode (>30 GHz), not achievable in the experimental method due to large losses in the power of the microwave current injected into the sample.^62^ For this reason, although the exact value of *J* is undeterminable for *t*_*Pt*_ < 1.36 *nm*, we just kept the value *J* = 5.5 mJ/m^2^ for simulations. This *J* magnitude is marked as a horizontal dashed line in Figure 5d. Its real value may reach any point from the hatched region, and particularly, it may follow the exponential dependence, as predicted in ref (59) and shown in Figure 5d too. For the thicker Pt (*t*_Pt_ ≥ 1.36 nm), the fitting procedure to the Pt polarization model returned the Pt polarization depth parameter ξ ≈ 0.47 nm, which is 1.5 times greater than ξ reported for the Py/Pt/Py structure.^59^

Summarizing, we emphasize that the coupling strength correlates with the regions from I to IV. The constant value of *J* within region I corresponds to large and undetectable coupling, whereas in region II, *J* is still significant and measurable. The intermediate region III is characterized by weak coupling, while samples within region IV are practically decoupled.

Having the magnetic parameters derived from the macrospin simulations of the spin diode FMR dynamics, we calculated longitudinal static magnetoresistance (*R*_*xx*_) dependencies on the external magnetic field in *H*_*x*_, *H*_*y*_, and *H*_*z*_ directions. We also modeled the anomalous Hall resistance (*R*_*xy*_) when the external magnetic field is applied in the *z* direction.

### 4.2 Magnetoresistance and Anomalous Hall Effect

The Pt-based magnetic multilayers are expected to exhibit a large spin magnetoresistance due to substantial spin–orbit interaction within the HM layer. These interactions cause the relatively large spin currents to be generated and injected into the ferromagnetic layers. The spin currents and spin accumulations at the Co/Pt interfaces influence the magnetoresistance of the sample, as predicted by the theoretical model presented in Section 3.2. Here, we focus on the spin–orbit interactions that are reflected in SMR. The SMR is defined as the difference in the longitudinal resistance measured in the saturated magnetization of Co layers under the external magnetic field applied in the *y* and *z* directions, i.e., SMR = *R*_*xx*_(*H*_*y*_) – *R*_*xx*_(*H*_*z*_), while the AMR is defined similarly as in Section 3.2 as AMR = *R*_*xx*_(*H*_*x*_) – *R*_*xx*_(*H*_*z*_).^41^ Also, we measured the AHE configuration (*R*_*xy*_) in the field applied in the *z* direction. All magnetoresistance and AHE measurements were performed by the DC current method sweeping the external magnetic field up to 10 kOe.

Then, we modeled the magnetoresistance dependencies with the use of the macrospin model. We used the parameters derived previously by fitting the model to the FMR experimental results (shown in Figure 5). For the sake of simplicity, we treat the considered sample as doubled bilayers: Co/Pt and Pt/Co. It allows us to calculate the resistance of the Co/Pt/Co structure as the equivalent resistance of layers connected in parallel:  where composite layer resistances are described by *R*_*xx*1(2)_ = *R*_0,1(2)_ + Δ*R*_*AMR*_*m*_*x*1(2)_^2^ + Δ*R*_*SMR*_*m*_*y*1(2)_^2^.^41^ The AHE-related resistances are given by *R*_*xy*_ = Δ*R*_AHE_*m*_*z*1(2)_.^41^ In Figure 6, we show the typical MR curves for samples from regions I to IV.

**Figure 7:**
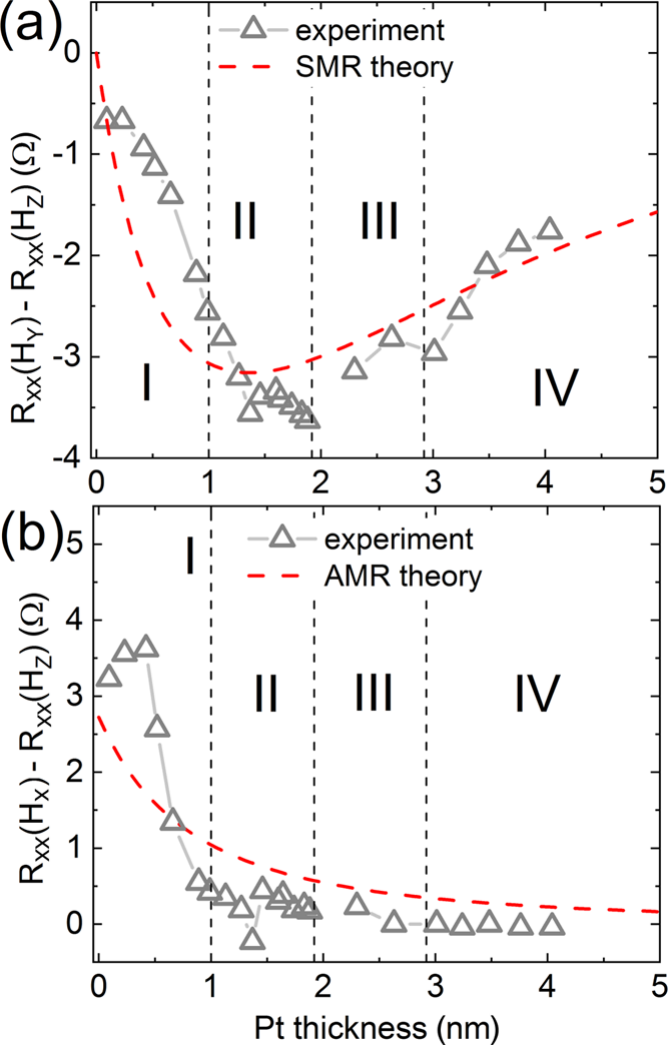
(a) SMR and (b) AMR amplitudes derived directly from the measurements (empty gray triangles) and calculated within the diffusive model (red-dashed lines) as a function of the Pt thickness.

For *t*_Pt_ = 0.52 nm, the macrospin qualitatively reproduces a narrow peak in MR. It also accounts for a more complicated dependence of *R*_*xx*_(*H*_*z*_) (see Figure 6a). The AHE curve does not exhibit a hysteresis and its shape is typical for the hard-axis rotation of both Co layers magnetized in-plane in the remanent state. The hysteresis in *R*_*xx*_(*H*_*z*_) is due to a competition between different anisotropies: in-plane and perpendicular that affect how the magnetizations rotate. Moreover, as one can see in Figure 7b, the AMR effect dominates in region I with the thinnest Pt layers.

On the contrary, in region II, the SMR is the highest as predicted by the spin-diffusive model and macrospin simulations (cf. Figure 7a). The representative sample from region II (*t*_Pt_ = 1.74 nm) exhibits a parabolic-like *R*(*H*_*y*_) dependence. It means that two Co layers are magnetized perpendicularly to the sample plane in the remanent state. Therefore, the AHE reveals a clear switching-like shape (see the inset in Figure 6b). Both, simulation and experimental results, show negligible contribution of the AMR.

For the sample from region III (*t*_Pt_ = 2.63 nm), the *R*(*H*_*y*_) is rather convex-shaped than parabolic. On the other hand, the AHE curve still exhibits a switching-like behavior. It suggests that the magnetization of layer 2 is tilted away from the perpendicular toward the in-plane direction.

For the thickest Pt layer (e.g., *t*_pt_ = 3.76 nm in Figure 6d) when the Co layers are weakly coupled (region IV), one can see *R*_*xx*_(*H*_*y*_) having a parabolic-like shape in high magnetic fields. This part of the curve is due to the rotation of the perpendicularly magnetized Co layer from the *z* to *y* direction. On the contrary, at low fields, there is a characteristic sharp peak related to the rotation of the in-plane magnetized Co layer from its remanent state direction to the y direction. The dependence was well reproduced by the macrospin model (black solid line in Figure 6d). The same macrospin parameters provide the satisfactory agreement of AHE magnetoresistance with experimental points (see the inset in the same figure). The AHE curve exhibits a smooth-edged shape hysteresis, characteristic for the simultaneous rotation of the bottom Co layer magnetization (*M⃗*_2_) in its hard direction and switching of the top Co layer between two states: ±*M⃗*_1,*z*_.

We supported the macrospin model with micromagnetic simulations in the case of the almost decoupled Co layers. The relevant calculations were performed with MUMAX3,^63^ where the LLG equation was integrated numerically for each simulation cell. Due to memory and time usage limits, the simulated area was nominally restricted to 5 × 20 μm^2^. However, we also utilized periodic boundary conditions along the *x* direction to produce a demagnetization tensor matching the actual experimental conditions. To optimize simulation performance, the cell size was chosen as 4.88 × 4.88 × 0.87 nm^3^ for *t*_Pt_ = 1.74 nm and as 4.88 × 4.88 × 0.94 nm^3^ for *t*_Pt_ = 3.76 nm. In both cases, the external magnetic field *H*_*y*_ was increased with a 500 Oe step, and the magnetization of Co layers was allowed to relax fully before moving to the next step. Then, the averaged magnetization vector for each layer was registered and used as an input for further resistance calculations. The micromagnetics revealed the same shapes of *R*_*xx*_ curves as the macrospin model, for the same parameters (or very close), as shown in Figure 5 (see the caption of Figure 6 for details). The agreement between macrospin and micromagnetic simulations confirmed that the macrospin parameters are reliable.

For the sake of completeness, in Figure 7, we show the Δ*R*_AMR_ and Δ*R*_SMR_ that were derived in the whole range of the Pt thickness from experiment and predicted by the spin-diffusive model described in Section 3.2. The obtained amplitudes agree to a satisfactory extent. As one can see from eqs 8 and 9, the SMR and AMR depend on the charge current flowing in HM and F layers, respectively. However, the currents in the HM layer are also influenced by spin accumulation at interfaces of this layer due to inverse SHE. The spin accumulation is mainly determined by the mean spin diffusion length (λ_HM_) and spin Hall angle (θ_SH_). The negative value of the SMR reaches its maximum at *t*_Pt_ ≈ 1.5 nm and decreases for thicker Pt layers, for which the spin decoherence affects the spin current, which, in turn, reduces the effective spin accumulation at the F/HM interface. On the contrary, the positive value of the AMR rapidly and monotonically decreases with HM thickness since the average charge current density flowing into the F layer decreases for the thicker Pt layer. Discrepancies between the experimental and theoretical MR dependencies in region I result from strongly mixed and alloyed interfaces for small thicknesses of Pt.

### 4.3 Spin Hall Angle and Spin–Orbit Torques

**Figure 8:**
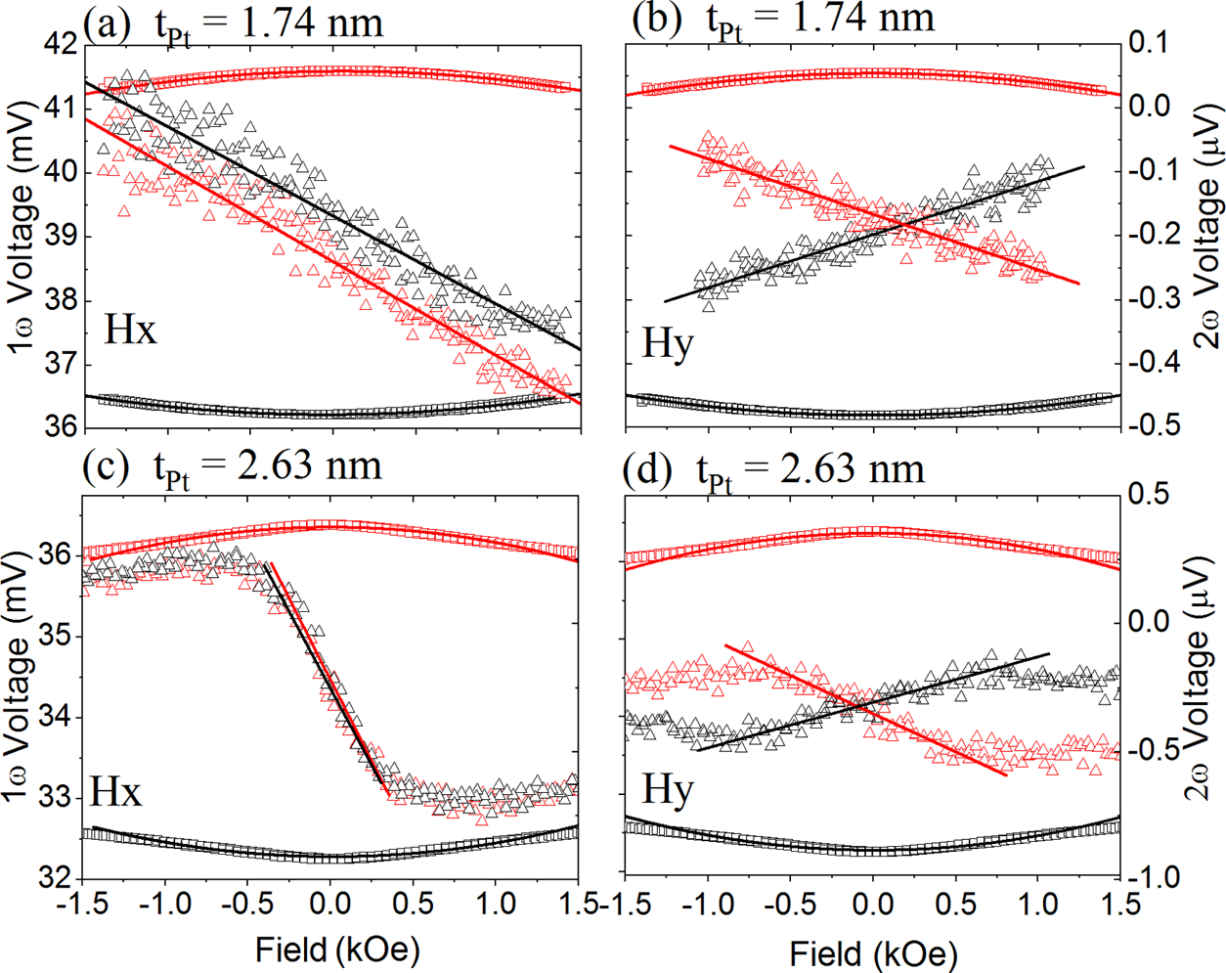
Experimental harmonic voltages *V*_ω_ and *V*_2ω_ for the representative samples from regions (a, b) II (*t*_Pt_ = 1.74 nm) and (c, d) III (*t*_Pt_ = 2.63 nm), both measured at the in-plane magnetic fields (*H*_*x*_ and *H*_*y*_) swept from −1.5 to +1.5 kOe (cf. eq 16). The fitted linear and quadric functions correspond to the field-sweeping method (see ref (65)).

**Figure 9:**
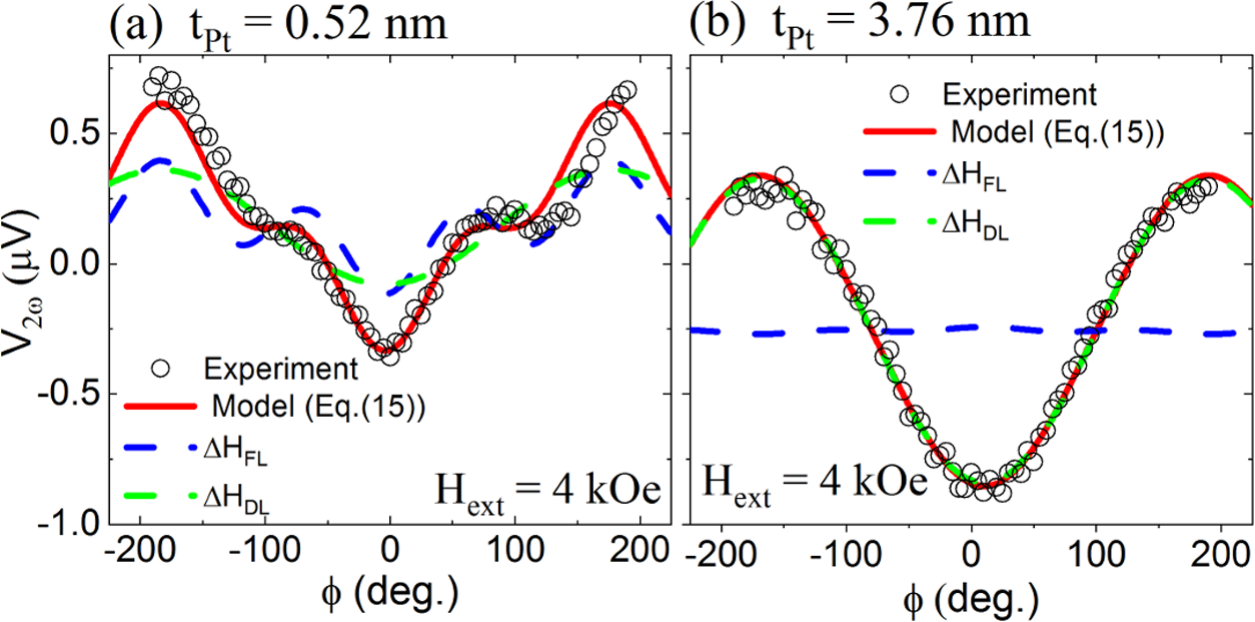
Experimental harmonic voltages *V*_2ω_ for the representative samples from regions (a) I (*t*_Pt_ = 0.52 nm) and (b) IV (*t*_Pt_ = 3.76 nm), both measured using the angular harmonic voltage method (cf. eq 17). The fitted trigonometric functions follow eq 17.

To quantitatively characterize the spin–orbit interactions in the Co/Pt/Co trilayers, we performed the harmonic measurements briefly described in Section 6.4. For the samples for which both or one of the Co layers is magnetized in-plane (regions I and IV) in the remanent state, we applied the angular harmonic voltage measurement method.^11,64^ On the contrary, in the case of the Co layers that magnetizations are perpendicularly oriented (regions II and III), we measured the field dependence of the relevant harmonic voltages.^65^ In the latter method, the damping-like (DL) and field-like (FL) components of SOT fields are determined using the following formula
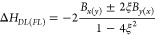
16where  and *H*_*x*(*y*)_ stands for the in-plane external magnetic field applied in the *x*(*y*) direction (cf. Figure 1). The parameter  is planar to the anomalous Hall effect ratio. The first and second harmonic voltages (*V*_ω_ and *V*_2ω_) measured as a function of the applied magnetic fields (*H*_*x*_ and *H*_*y*_) are plotted in Figure 8. The results shown in Figure 8 are representative of the samples from regions II (*t*_Pt_ = 1.74 nm) and III (*t*_Pt_ = 2.63 nm). Next, we could use eq 16 and follow the method described in ref (65) to calculate SOT effective fields Δ*H*_*DL*(*FL*)_ in samples from regions II and III. Nevertheless, the above method turns out to be ineffective in the case of samples with one or both layers magnetized in-plane. In this case, to determine Δ*H*_DL(FL)_, we measured the angular dependence of *V*_2ω_ on the magnetic field applied in the sample plane. Such a dependence may be expressed as follows^11,64^
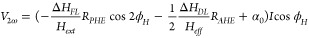
17where ϕ_*H*_ stands for the in-plane angle of the magnetic field. The term α_0_ is the anomalous Nernst effect (ANE) coefficient due to thermal gradients within the samples induced by the Joule heating.^64^ The experimental angular dependencies *V*_2ω_(ϕ_*H*_) for the samples with *t*_*Pt*_ = 0.52 nm (region I) and *t*_*Pt*_ = 3.76 nm (region IV) are shown in Figure 9a,b. As one can see, the damping-like SOT effective field (Δ*H*_*DL*_) is proportional to the cosϕ_*H*_, whereas the field-like one (Δ*H*_*FL*_) is proportional to the cosϕ_*H*_ cos 2ϕ_*H*_. Moreover, the , where *K*_*eff*_ (defined as in Section 4.1) and *M*_*S*_ are the parameters of the Co layers magnetized in-plane. As long as we knew the magnetic parameters (summarized in Figure 5) of the layers, we could fit eq 17 to the experimental data and consequently determine both field-like and damping-like SOT components.

**Figure 10:**
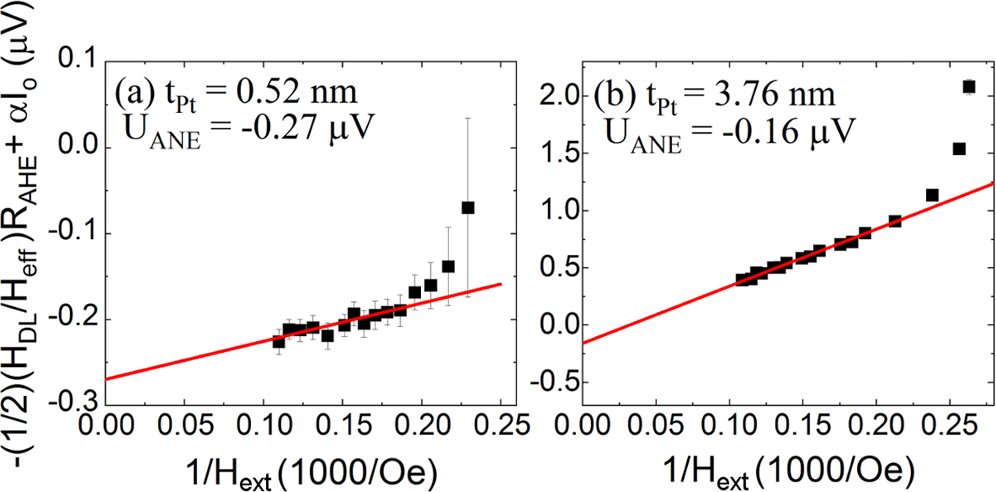
Amplitude of eq 17 plotted as a function of the magnetic field 1/*H*_ext_ measured in the samples with Pt thicknesses 0.52 nm (a) and 3.76 nm (b) at angle ϕ_*H*_ = 45°. The interceptions with the *y* axis indicate the ANE contribution.

In addition, by plotting the terms proportional to Δ*H*_*DL*_ as a function of 1/*H*_*ext*_, we could estimate the contribution of the ANE. One should note that the offset of linear fit (at 1/*H*_*ext*_ = 0) visible in Figure 10a,b is the ANE contribution α_0_*I*_0_. We show the relevant plots for two samples (*t*_*Pt*_ = 0.52 and 3.76 nm). The small offsets of fitted lines (≈ −0.3μ*V*) and (≈ −0.15μ*V*) corresponding to the ANE-related electric fields *E*_ANE_ ≈ −0.03*V*/*m* and *E*_*ANE*_ ≈ −0.015*V*/*m*, respectively, are much smaller than the values in the Co/Pt systems present in the literature, e.g., in ref (64). Therefore, it suggests that the ANE contribution is negligible in our devices with thin and thick Pt layers. It is worthy to notice that the dependencies shown in Figure 10 can be used to examine the applicability of eq 17. At low magnetic fields, the dependencies deviate from the linear and eq 17 is not fulfilled. On the other hand, according to the model, the dependencies are linear at high fields.

**Figure 11:**
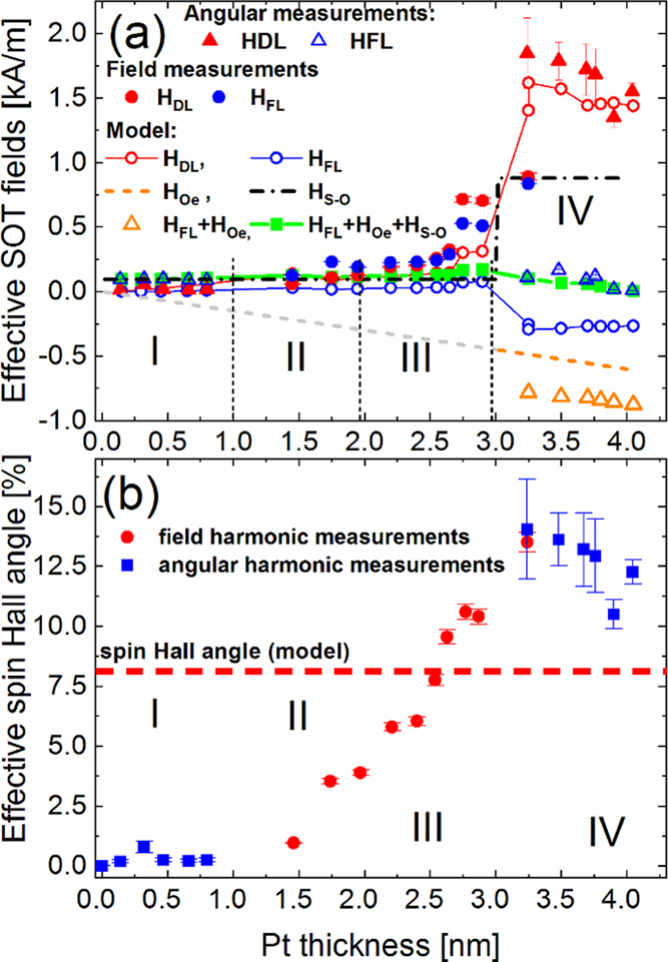
(a) Experimental SOT effective fields: damping-like and field-like components obtained with macrospin magnetic parameters from Figure 5 (blue- and red-filled points and blue empty triangles), the effective theoretical sum of SOT fields: *H*_FL_ (blue empty points), and separately *H*_DL_ (red empty points) acting on two Co layers. The amplitude of the Oersted field (*H*_Oe_) (orange and gray dashed lines) and the Rashba–Edelstein spin–orbital field (*H*_S–O_) (black dash-dotted line). The resultant field-like SOT (filled green squares) including the *H*_S–O_ field in regions I–IV and, additionally, the Oersted field in region IV. Orange triangles correspond to the case when the Oersted field only is added to the *H*_*FL*_. (b) The effective spin Hall angle is determined from field (red circles) and angular (blue squares) harmonics measurements, with the *H*_DL_ amplitudes from panel (a). The red-dashed line indicates the theoretical value of the spin Hall angle fitted to the experimental MR dependencies (cf. Figure 7).

We summarize the results for the samples from all regions (I–IV) in Figure 11a. For very thin Pt (region I), both field-like and damping-like components are small, although the former contributes slightly more than the latter one. In regions II and III, both components increase in their magnitudes; however, in region III, the DL component (filled red points), due to intermixing and alloying Co and Pt, surpasses the FL component’s (blue-filled points) magnitude. The most intriguing is region IV, where the damping-like component dominates over the field-like, especially for *t*_*Pt*_ > 3 nm. For such a thick Pt layer, the *H*_*DL*_ saturates, while the *H*_*FL*_ drops again toward small values. Similarly, the effective spin Hall angle defined by  starts to increase in region II where the Pt thickness is sufficient to generate significant spin currents and consequently the *H*_*DL*_ SOT field. The θ_*SH,eff*_ continues increasing in region III and then reaches its maximum value c.a. 14% at *t*_*Pt*_ = 3.24 nm. Next, it slightly decreases with the Pt thickness in region IV.

One should note that θ_*SH,eff*_ accounts for the spin accumulation effect at interfaces in the trilayer system. For this reason, the experimental value of θ_*SH,eff*_ may substantially differ from the theoretical one (θ_*SH*_ in Section 3.2), introduced as a material parameter of pure SHE efficiency at a very thick HM thickness limit. More precisely, the effective spin Hall angle is smaller than the theoretical one (θ_*SH*_) for *t*_*Pt*_ < 2.6 nm. For thicker Pt layers, θ_*SH,eff*_ increases and becomes higher than the theoretical value. It is correlated with the change of magnetization direction from perpendicular to the in-plane for the thick Pt layer, particularly in region IV. To shed light on the θ_*SH,eff*_ dependence on Pt thickness, one needs to go back to Figure 11a based on which the θ_*SH,eff*_ was determined. The two modes of the experimental harmonics method allow the measurement of the effective SOT fields acting on the Co layers magnetized only in the plane (angular method) or perpendicular to the plane (field method). This limitation arises from very high fields required to saturate the layers magnetized in-plane (perpendicularly) at remanence into the perpendicular (in-plane) direction. Therefore, the data recorded with the experimental harmonic method accounts for both magnetic layers when magnetized in the same direction. That is the case of samples from regions I–III. On the contrary, only one magnetic layer can be sensed by the measurement setup in region IV, where both magnetizations are orthogonal. Therefore, the experimental conditions differ in regions I–IV. As a consequence, the experimentally determined *H*_*DL*_ and *H*_*FL*_ fields in regions I–III are the sums of the related SOT fields acting on F1(top) and F2(bottom) magnetic layers. Since the spin currents generated by the SHE have opposite polarizations at F/HM and HM/F interfaces, the SOT effective fields also have the opposite signs. The residual fields, defined as *H*_*DL*_ = *H*_*DL*,1_ + (−*H*_*DL*,2_) and *H*_*FL*_ = −*H*_*FL*1_ + *H*_*FL*2_, come from the different interface properties, included in real parts of mixing conductances *G*_*r*_^(1)^ and *G*_*r*_^(2)^ of the diffusive model (cf. Section 3.2). To support our analysis, we calculated the SHE-induced SOT effective fields using the formulas (eqs 14 and 15) and magnetic parameters from Figure 5. Next, we plotted the difference of *H*_*DL*,1_ and *H*_*DL*,2_ in regions from I to III at the experimental thicknesses of Pt. Nevertheless, considering the experimental conditions discussed above, we plotted the *H*_*DL*,2_ field only in region IV. The results are shown in Figure 11a as red empty circles and agree with the experimental ones (red-filled points, triangles, and circles) to a satisfactory extent.

On the contrary, a similar procedure is insufficient to reproduce the experimental *H*_FL_ field. The theoretical *H*_*FL*_ (empty blue circles) depends differently on the HM thickness than the experimental one (blue-filled circles and empty blue triangles). However, the diffusive model does not account for an Oersted field (*H*_Oe_) coming from the charge current, as well as a spin–orbital field (*H*_S–O_) at interfaces, arising from the REE. Both fields have the same direction as SHE-induced *H*_*FL*_. Thus, the additional terms have to be included in the analysis of the effective field-like field. We assumed that the Oersted field linearly increases with the Pt thickness (gray and orange dashed lines in Figure 11a), whereas the *H*_*S–O*_ (black dash-dotted line) is independent of the HM thickness.^19,42^ Since the Oersted field has the same amplitude with opposite signs in both F layers, its impact on the resultant *H*_*FL*_ cancels out and does not affect the field-like SOT in I–III regions. For the same reason, as discussed above, the *H*_*Oe*_ adds to *H*_*FL*_ in region IV (see the full orange triangles in Figure 11a). Conversely, the *H*_*S–O*_ fields do not cancel out due to the difference in F/HM and HM/F interfaces. Therefore, their difference contributes to the total *H*_*FL*_ term in regions I–III. In region IV, the amplitude *H*_*S–O*_ significantly increases because the perpendicularly magnetized Co layer is out of the experimental detection. Thus, the estimated magnitude generated at the Co/Pt interface was 0.86 kA/m. One should note that such a substantial value of the SOT field due to REE is much higher than its FL counterpart coming from the SHE. Moreover, it is of the order of the charge-current-induced effective Oersted field. However, all fields of SHE-related *H*_*FL*_, *H*_*Oe*_, and *H*_*S–O*_ must be considered in the total FL SOT component to achieve a satisfactory agreement with experimental data in the F/HM/F trilayer system (see the solid green line in Figure 11a). The discrepancies at the border between regions III and IV are due to the unreliability of the experimental methods (field and angular harmonics) applicable only when magnetic layers are magnetized fully in the plane or fully perpendicularly to the plane. This requirement is not fulfilled in the intermediate case, especially at *t*_*Pt*_ = 2.87 nm. It has its consequence in the spin Hall angle value that slightly drops at this Pt thickness. On the other hand, at *t*_*Pt*_ = 3.24 nm, the SOT fields determined from the field and angular harmonics methods differ. In this case, the field harmonics method detects the magnetic layer magnetized perpendicularly. Therefore, as discussed earlier, the SOT fields at the Pt/Co interface are different from those at the Co/Pt interface. However, the spin Hall angle (Figure 11b) treated as the HM material parameter is the same for both experimental methods. For thicker Pt, the detection signal in the field harmonics measurement was too weak to properly determine the *H*_*DL*_ and *H*_*FL*_ effective fields.

## 5 Conclusions

The paper presents the detailed results on structural, static, and dynamic properties of the Co/Pt/Co trilayer in which the Pt features a double role of the source of spin currents and interlayer exchange coupling in a wide range of the Pt layer thickness. First, we showed the Co/Pt and Pt/Co inherent interface asymmetries that resulted in different interfacial spin–orbit-related properties of ferromagnetic layers, magnetic anisotropies, and the effective spin–orbit torque fields due to SHE and REE. The difference in anisotropies makes the Co layer with stronger perpendicular anisotropy be a primary layer that determines the magnetization direction of the secondary Co layer through the interlayer exchange coupling. Therefore, we were able to determine four ranges of Pt thickness where the trilayer reveals different static and dynamic behaviors correlated with the strength of coupling: region I (two Co magnetizations are in-plane), region II (Co magnetizations are both perpendicular to the plane), region III (one of the Co magnetizations is tilted from the perpendicular direction), and, finally, region IV (one Co magnetization is in-plane, whereas the second one is perpendicular). We showed that the experimental relation of dispersions and magnetoresistances differs in each region. This difference is accounted for by the macrospin models that we used, and therefore, both the experimental magnetoresistance and SOT–FMR relations of dispersion were reproduced by theoretical calculations to a satisfactory extent. Moreover, we made a detailed analysis of the SOT effective fields determined using harmonics measurements. We showed that the experimental method applied to trilayers with two Co magnetizations aligned both in-plane or both out-of-plane allows measuring a difference of the effective SOT fields coming from two F/HM and HM/F interfaces. However, when two magnetizations are orthogonal, the experimental technique enables measuring SOT fields from the single F layer. The experimental results revealed this feature and were successfully parameterized with the magnitudes of damping-like and field-like SOT obtained from the diffusive model. Finally, both experimental and theoretical data allowed us to determine the contribution of Oersted (*H*_*Oe*_) and spin–orbital (*H*_*S–O*_) fields to the resultant experimental field-like SOT. We showed that the latter contribution due to REE might be comparable to the effective Oersted field and more significant than the field-like SOT caused by the SHE.

## 6 Experimental Methods

### 6.1 Sample Preparation

The base pressure in the deposition chamber was 4.5 × 10^−8^ mbar. The substrate temperature was at room temperature (RT). The Ar pressure during the deposition process was 8.53 × 10^−3^ mbar, except for the deposition of the MgO layer when it was 8.52 × 10^−3^ mbar. A fixed direct current (DC) power of 8 W for Pt and 15 W for Co and an alternating (RF) power of 75 W for MgO and 50 W for Ti were used. The Pt layer was deposited in a wedge-shaped form with thickness varying from 0 to 4 nm along a 20 mm long sample edge (*x* coordinate). The resulting thickness gradient (0.0002 nm/μm) was achieved by the controlled movement of a shutter. Thicknesses of all layers were determined from the deposition growth rate of particular materials calibrated using X-ray reflectivity measurements. Before patterning to the form of bar devices, all as-deposited samples were characterized by X-ray diffraction θ–2θ (XRD) and grazing incidence diffraction (GIXD) and also examined by the polar Kerr magnetometer (p-MOKE) and time-resolved TR-MOKE to determine the static and dynamic magnetization parameters, which studies have been described in detail in a separate work.^66^ After basic characterization of continuous samples, multilayers were patterned using optical direct imaging lithography and ion etching to create a matrix of Hall- and resistance-bar devices, with different thicknesses of Pt for subsequent electrical measurements. The sizes of prepared structures were 100 μm × 20 μm for magnetoresistance and spin diode effect measurements, whereas they were 100 μm × 10 μm for the AHE and harmonics measurements. The sizes of the devices assure that the structure symmetry is broken in the direction perpendicular to the layer plane only, and therefore the effects of REE-related fields and spin current gradients can be neglected.

Al(20)/Au(30) electrical leads of 100 μm × 100 μm were deposited in a second lithography step followed by the lift-off process.

### 6.2 Resistance Measurements

Specific locations of pads near the Hall bars were designed for measurement in a custom-made rotating probe station, allowing a 2- or 4-point measurement of electrical transport properties in the presence of the magnetic field applied at an arbitrary azimuthal and polar angle with respect to the Hall bar axis. The scheme of the experimental setup for longitudinal (*R*_*xx*_) and Hall (*R*_*xy*_) resistance measurements is shown in Figure S1. The resistance was measured using a four-point method,^67^ and resistivities of Pt and Co layers were determined using a parallel resistor model and the method described by Kawaguchi et al.^68^ The Pt and Co resistivity analyses yielded 59 μΩ cm and 72.5 μΩ cm, respectively.

### 6.3 Spin Diode Effect Measurements

The magnetic dynamics of the patterned samples was electrically detected with the FMR measurements through the spin diode effect.^57^ The scheme of the measurement setup is shown in Figure S2. The effect occurs when the rf current flows through the magnetoresistive element that in the case of our samples exhibits the anisotropic magnetoresistance (AMR) and SMR. Then, the current-related effective magnetic fields (as Oersted, *H*_*Oe*_, or SOT fields) force the sample magnetization to oscillate. The magnetization oscillations, in turn, result in the time-dependent resistance of the sample, which mixes up with the rf current.

Therefore, the measured output voltage may be expressed as *V*_*out*_ = *I*_0_ cos (ω*t*) ·*R*(ω*t* + Φ), where the Φ is the phase shift between the current and resistance. One notes that *V*_*out*_ includes ac and dc contributions, namely, *V*_*out*_ = *V*_*dc*_ + *V*_*ac*_= *I*_0_ δ *R* cosΦ + *I*_0_ δ *R*(2 ω *t* + Φ). The dc output voltage depends on the angular frequency, external magnetic field, and parameters of the sample.

The spin diode FMR measurements are performed with an amplitude-modulated radiofrequency (rf) current with a corresponding power of P = 16 dBm and frequencies ranging from 1 to 25 GHz. The mixing voltage (*V*_*out*_) is measured using a lock-in amplifier synchronized to the rf signal. The in-plane magnetic field (*H*_*ext*_) is applied at ϕ = 45 deg with respect to the microstrip long axis and was swept from 0 up to 9 kOe.

### 6.4 Harmonic Hall Voltage Measurements

To determine spin–orbit torque fields (damping- and field-like components), as well as the spin Hall angle, we used the methods based on the harmonic measurements.^11,64,69^ For these measurements, we apply a low-frequency constant-amplitude sinusoidal voltage to the Hall bar device with current density from j= 3.12 × 10^10^ A/m^2^ to *j* = 3.29 × 10^10^ A/m^2^ depending on the Pt layer thickness. Using two lock-in amplifiers, we measure simultaneously the in-phase first harmonic (*V*_ω_) and the out-of-phase second harmonic Hall voltages (*V*_2ω_) as a function of an external magnetic field *H*_*ext*_. The sample is rotated within the x–y plane, making an azimuthal angle ϕ_*H*_ with the x-axis, as depicted in Figure S3. The measurements were conducted in two configurations: the first one is referred to as field measurements and the samples were probed with the different magnitudes of the external magnetic field applied along both the x and y directions,^65^ while the second configuration is the angular measurements. The sample is rotated in the x–y plane while the *V*_ω,2ω_ is recorded^64,65^ for fixed magnitudes of the external magnetic field. The field measurements are relevant in the case of samples with out-of-plane effective anisotropies. On the contrary, the angular measurements allow us to detect harmonic signals in samples with in-plane effective anisotropy.

## Supporting Information Available

Experimental setups for electrical measurements, the in-plane anisotropy magnitudes, and the perpendicular anisotropy easy-axis deviation angles (PDF)
