# Supplementary material for: Study of Spin–Orbit Interactions and Interlayer Ferromagnetic Coupling in Co/Pt/Co Trilayers in a Wide Range of Heavy-Metal Thickness
Source: ACS Appl Mater Interfaces. 2021 Sep 24;13(39):47019–32. doi: 10.1021/acsami.1c11675 (PMC8519406; doi:10.1021/acsami.1c11675)
Supplement: Supplementary file 1 — am1c11675_si_001.pdf [file am1c11675_si_001.pdf]

Supporting Information for "Study of Spin-Orbit Interactions and Interlayer Ferromagnetic Coupling in Co/Pt/Co Trilayers in Wide Range of Heavy Metal Thickness"

Piotr Ogrodnik,[*,†,§] Krzysztof Grochot,[†,∥] Łukasz Karwacki,[‡,⊥] Jarosław Kanak,[†] Michał Prokop,[¶] Jakub Chęciński,[†] Witold Skowroński,[†] Sławomir Ziętek,[†] and Tomasz Stobiecki[†,∥]

†*Institute of Electronics, AGH University of Science and Technology, 30-059 Kraków, Poland*

‡*Institute for Theoretical Physics, Utrecht University, Princetonplein 5, 3584 CC Utrecht, Netherlands*

¶*Catalan Institute of Nanoscience and Nanotechnology (ICN2), CSIC and BIST, Campus UAB, Bellaterra, 08193 Barcelona, Spain*

§*Faculty of Physics, Warsaw University of Technology, 00-662 Warsaw, Poland*

∥*Faculty of Physics and Applied Computer Science, AGH University of Science and Technology, 30-059 Kraków, Poland*

⊥*Institute of Molecular Physics, Polish Academy of Sciences, ul. M.Smoluchowskiego 17, 60-179 Poznań, Poland*

E-mail: piotr.ogrodnik@pw.edu.pl

1 Experimental setups for electrical measurements

Below we present the experimental setups used for the transport measurements (MR, AHE) as well as the electrically detected spin-diode SOT-FMR.

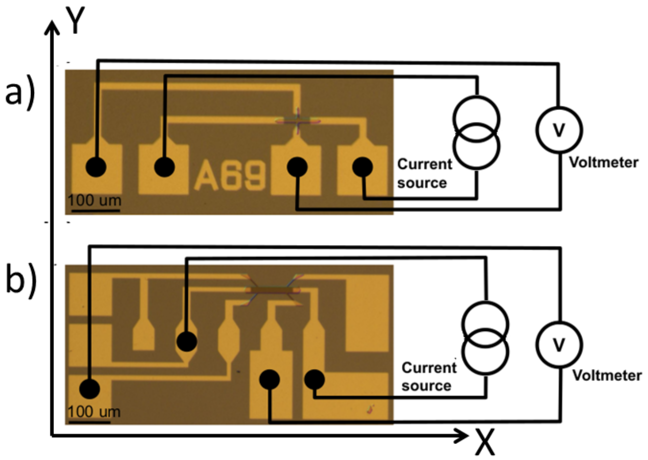

Fig. S1: The Hall resistance (R_{xy}) (a) and 4-points longitudinal magnetoresistance (b) measurement setups depicted on micrographs of the devices.

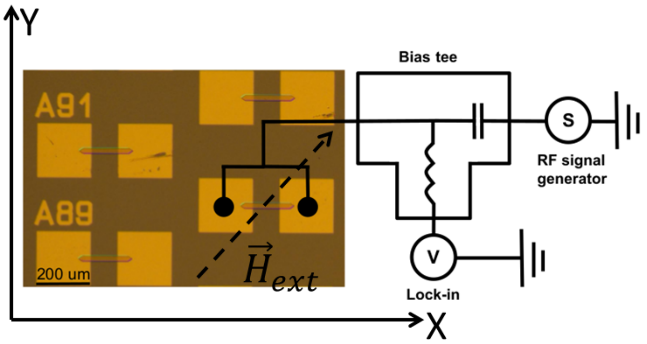

Fig. S2: The resistance-bar attached to the spin-diode FMR measurement setup (see main text for details) depicted on the set of four patterned devices photomicrography. The dashed arrow indicates the direction of the external magnetic field $\vec{H}_{ext}$.

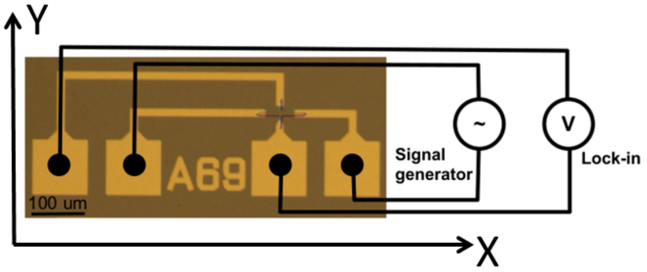

Fig. S3: The Hall-bar and the experimental setup for harmonic Hall voltage ($V_{\omega,2\omega}$) measurements.

2 Anisotropies

In fitting procedure we used a relatively small in-plane anisotropies with the maximum value of 0.025 MJ/m3. The in-plane anisotrpoy improves the MR fitting, especially in the region I.

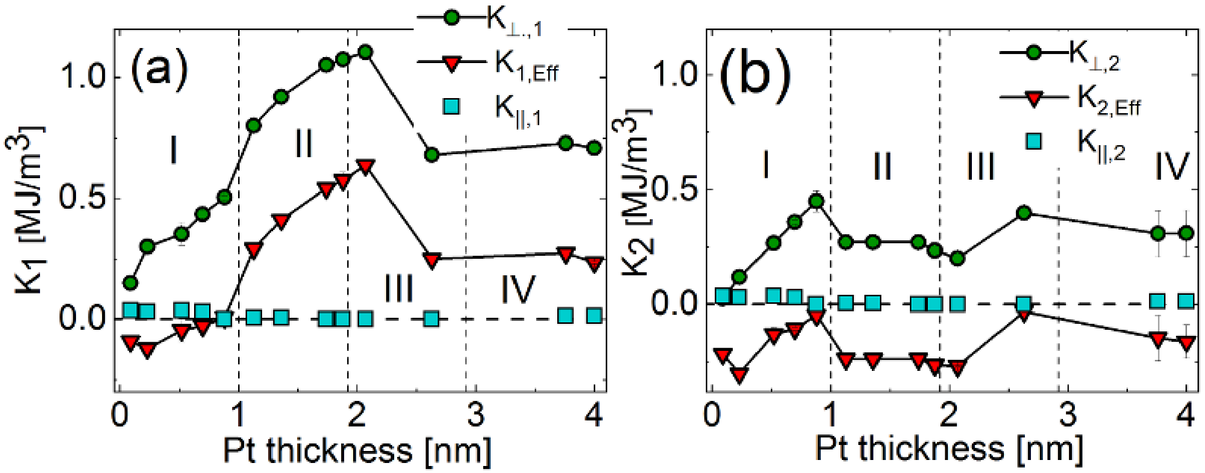

Fig. S4: The in-plane anisotropies ($K_{\|,1,2}$) compared to the perpendicular ones. The anisotropies are estimated from the fitting macrospin model to the experimental data

The perpendicular anisotropy easy axes were deviated from the z direction by a maximum angle of 30 deg. For the definition of the anisotropy deviation angles see Figure below.

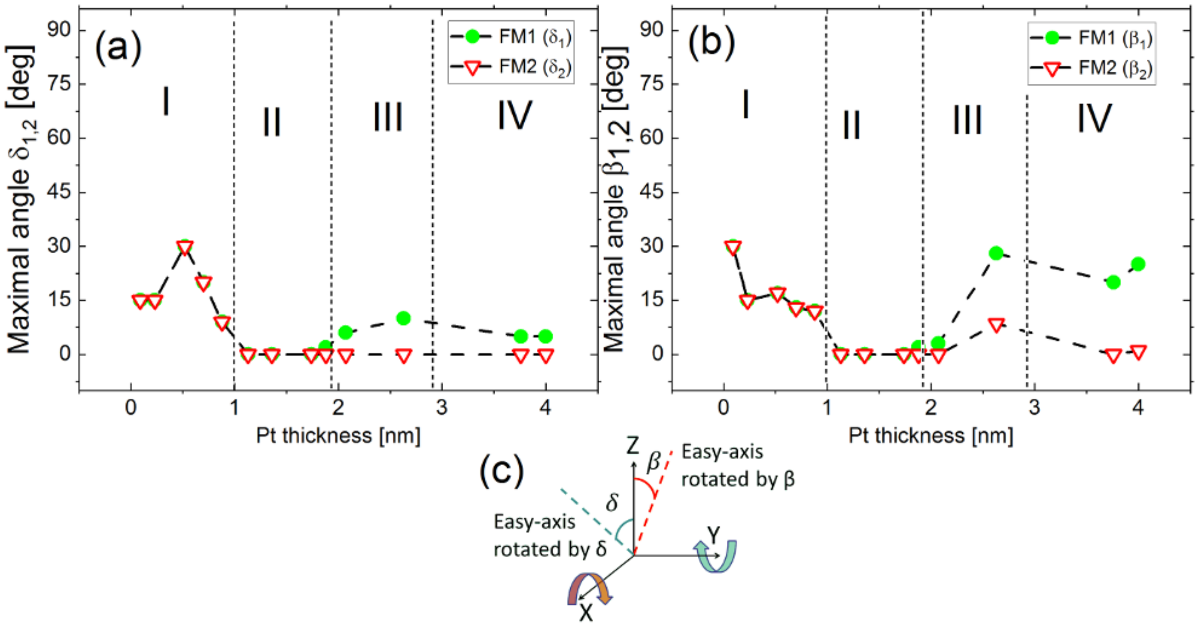

Fig. S5: The perpendicular anisotropy easy-axis deviation angles (a) δ and (b) β dependencies on the Pt thickness. (c) the rotation of easy-axis around x and y axes.
